# Improved Balance and Gait Ability and Basic Activities of Daily Living after Comprehensive Geriatric Care in Frail Older Patients with Fractures

**DOI:** 10.3390/healthcare9050560

**Published:** 2021-05-11

**Authors:** Marco Meyer, Stefanie Schmetsdorf, Thomas Stein, Ulrich Niemoeller, Andreas Arnold, Iris Reuter, Karel Kostev, Ralf-Achim Grünther, Christian Tanislav

**Affiliations:** 1 Department of Geriatrics and Neurology, Diakonie Hospital Jung Stilling, 57074 Siegen, Germany; Marco.Meyer@diakonie-sw.de (M.M.); Stefanie.Schmetsdorf@diakonie-sw.de (S.S.); Thomas.Stein@diakonie-sw.de (T.S.); Ulrich.Niemoeller@diakonie-sw.de (U.N.); andreas.arnold88@gmx.de (A.A.); 2 Department of Neurology, Justus Liebig University, 35392 Giessen, Germany; Iris.Reuter@neuro.med.uni-giessen.de; 3 Epidemiology, Philipps University Marburg, 35037 Marburg, Germany; karel.kostev@iqvia.com; 4 Department of Surgery, Diakonie Hospital Jung Stilling, 57074 Siegen, Germany; Ralf-Achim.Gruenther@diakonie-sw.de

**Keywords:** geriatric care, frailty, comprehensive geriatric care, older patients

## Abstract

(1) Purpose: Comprehensive geriatric care (CGC) is a multidisciplinary treatment approach for elderly patients. We aimed to investigate outcomes in fracture patients who had been treated using this approach in a large geriatric unit. (2) Methods: This observational cohort study assessed the gait function (using the Tinetti Balance and Gait Test (TBGT)) and basic activities of daily living (ADL) (using the Barthel index (BI)) before and after CGC and compared the results. Baseline data, walking ability assessments (Timed Up and Go, TUG), and cognitive status (mini mental status examination, MMSE) were also analyzed in the subgroup of patients with versus without fractures. (3) Results: Out of 1263 hospitalized patients, 1099 received CGC (median age: 83.1 years (IQR: 79.0–87.8 years); 64.1% were female). TBGT improvement was observed in 90.7% and BI increased in 82.7% of fracture patients. A TBGT improvement of >5 was noted in 47.3% and was associated with female sex, a lower BI at admission (median: 40 versus 45; *p* = 0.010), and poorer mobility on admission (TUG: median 5 versus 4; *p* = 0.001). An improvement in BI of ≥15 was observed in 63.0% of the cases, and was associated with a better cognitive status (MMSE: median 25 versus 18; *p* = 0.001) and inversely associated with diabetes mellitus and a previous stroke. (4) Conclusion: CGC in specialized geriatric units improves the balance and gait and the basic ADL in geriatric patients. After fracture, female patients are more likely to experience improvements in gait and balance, while patients with better cognitive condition are more likely to experience improvements in ADL.

## 1. Introduction

Specialized geriatric units are increasingly being integrated in the clinical care structure in Germany and other countries to cover the specific needs of elderly patients [1]. Comprehensive geriatric care (CGC) is an adapted treatment following predefined protocols in order to meet the requirements of elderly multimorbid patients [2,3,4,5]. CGC is characterized by a multidisciplinary approach for developing individual treatment strategies by a multidisciplinary team including different medical professionals such as physicians, occupational therapists, physiotherapists and speech therapists, psychologists, and social workers [6,7,8,9,10,11]. In addition to the medical treatment, the main goal of this interdisciplinary approach is to improve patients’ functional outcome in order to help them maintain their independence in the home environment [2,12,13,14,15]. The benefits of CGC in older hospitalized patients have been demonstrated in recent years [1]. Patients with medical, neurological and surgical diagnoses as well as those with fractures may benefit from CGC treatment [16,17,18,19,20,21]. The program is perhaps especially relevant for older patients, as fractures might cause relative immobility for the individual patient but a complete recovery could be achieved after functional bone restitution [17,22]. Therefore, it is of particular importance that older patients in this situation receive the appropriate treatment in order to ensure the best conditions for rehabilitation.

It is for this reason that the present study specifically aimed to investigate balance and gait ability and its impact on basic activities in daily living (ADL) in older patients with fractures who underwent CGC in a large geriatric unit and to identify factors that might influence outcomes.

## 2. Methods

### 2.1. Patients and Measures

All patients hospitalized between May 2018 and May 2019 in our 50-bed geriatric department were selected for the present analysis. These patients were referred to the geriatric unitby the emergency and other in-house departments, other hospitals or resident doctors. All relevant data concerning patients’ care and medical treatment are documented and recorded systematically and used regularly as a basis for interdisciplinary discussions, quality assurance measures and billing calculations. Both demographic parameters and information regarding patients’ morbidity and functional outcome were used for the current analysis: age, sex, medical co-morbidities, and information on short-term adverse events during the hospitalization. Data on balance and gait ability and basic ADL were also used for this analysis; we selected patients who had received comprehensive geriatric care.

### 2.2. Comprehensive Geriatric Care (CGC)

The selection criteria for the selection for CGC were:

- Age ≥ 65 years;
- Multimorbidity (two or more chronic diseases);
- Disabling deficits expected to improve after completing CGC.

All criteria needed to be fulfilled and were verified by an experienced geriatrician prior to admission. All patients allocated to the comprehensive geriatric care program underwent a structured assessment; patients’ mobility, ability to cope with daily tasks, cognitive function, and emotional and social conditions were documented upon hospital admission and again at discharge. The assessment included the Barthel index, Timed up and go test, Tinetti Balance and Gait test, the Mini Mental Status Assessment and the Geriatric Depression Scale, while patients’ social status was determined in structured interviews [23,24,25,26,27]. A personalized treatment plan was developed for each patient based on the results of the assessment on admission. The selected treatment was adapted to patients’ deficits and continuously re-evaluated by the therapeutic team and every patient received a minimum of 20 treatment units. Each treatment unit was at least 30 min long and consisted of one of the following treatment methods: physiotherapy, occupational therapy, speech therapy/orofacial therapy including the assessment of and therapy for swallowing, and psychological support. All procedures are summarized in Table 1. The interdisciplinary team—consisting of geriatric nurses, physiotherapists, occupational therapists, speech therapists, and psychologists—administered the CGC under the supervision of an experienced geriatrician. The program was complemented by daily medical visits and a weekly team conference to discuss treatment progress (with adaptions if necessary).

### 2.3. Assessment of Balance and Gait (Tinetti Balance and Gait Test, TBGT)

TBGT is a commonly used tool for assessing balance and gait dysfunction and fall risk in elderly patients. The balance is determined by examining the patient in a sitting and standing position, when rising from and sitting down in a chair, rotating 360°, and by applying slight pressure on the patient’s chest. The gait function is evaluated by reporting the length, height, symmetry, and continuity of the steps. Each item is worth 0–2 points for a maximum TBGT score of 28. The lower the TBGT score, the higher the risk of falling; mobility restrictions can be expected in patients with low scores [26]. In order to determine the outcome of CGC, we defined a relevant difference in TBGT of at least 5 points between admission and discharge. This gap was selected arbitrarily as an appropriate means of measuring clinically relevant differences. TBGT assessment after CGC was classified into three categories: unchanged, improved, and worsening.

### 2.4. Assessment of Basic Activities of Daily Living (Barthel Index, BI)

The BI is a widely used scoring system within the clinical routine for assessing patient disability. It includes ten different items (ingestion, bed/chair transfer, dressing, walking, grooming, climbing stairs, use of toilet, bathing, continence of bowels, and controlling bladder) concerning patients’ basic ADL and mobility. The examiner allocates values of between 0 and 100 for each item according to the patient’s ability; the higher the value, the better the functional status [24,28]. The cut-off point for clinically relevant BI improvements between hospital admission and discharge was determined at BI values ≥15. This gap was selected arbitrarily, as an appropriate means of measuring clinically relevant differences. We classified the shift in patients’ disability prior to and after CGC into three categories: unchanged, improved, and worsening.

### 2.5. Statistical Analyses

Data for continuous variables are expressed as median values and interquartile ranges. Categorical variables are reported as frequencies and percentages. The Kolmogorov-Smirnov one-sample test was used to verify normal distribution. Nonparametric data were analyzed by applying a two-tailed Mann-Whitney U-test, while Fisher’s exact test was used to compare relative frequencies. The SPSS software, (version 22.0, IBM Corporation, Armonk, NY, USA) was used for the statistical analyses.

### 2.6. Ethical Approval

We obtained ethical approval from the local ethics committee for the offline analysis of data obtained in the delivery of clinical care (protocol number: 2019-517-f-S).

## 3. Results

Out of 1263 patients hospitalized in our specialized geriatric unit, 1099 patients underwent CGC and were included in the analysis (median age: 83.1 years (IQR: 79.0–87.8 years); 64.1% were female). Patients with fractures (n = 300) were older than those without (n = 799) (median 85 years (IQR: 81.1–89.6 years); the subgroup of patients with fractures was predominantly female (73% versus 27%). We detected 168 (56%) patients with fractures of the lower extremities, while a further 45 patients (15%) had a fracture of the pelvic region and 42 (14%) suffered a fracture of the spinal column. A fracture of the upper extremities was detected in 25 patients (8.3%). A fracture of the thorax was identified in 8 patients (2.7%), while 12 patients (4%) presented with fractures in other locations. On admission, the median Barthel index was slightly lower in fracture patients than in non-fracture patients (40 (IQR: 30–50) versus 45 (30–60), *p* = 0.001). The distribution of co-morbidities, other baseline characteristics, and assessment results are summarized in Table 2.

**Figure 1:**
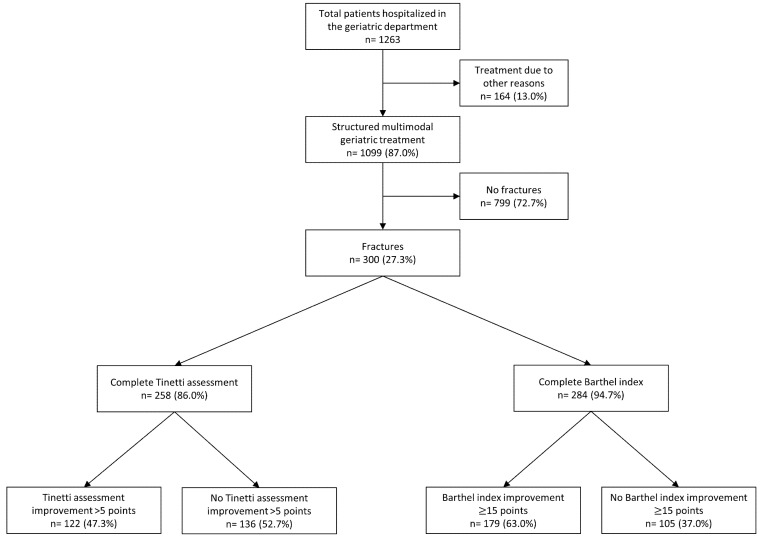
Patient selection.

**Figure 2:**
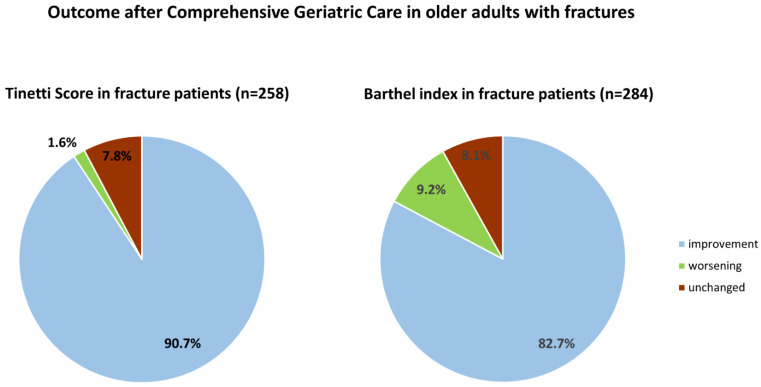
Fracture patient outcome after comprehensive geriatric care.

Out of 300 patients with fractures, 86.0% received a full TBGT assessment and 94.7% underwent the complete basic ADL assessment by BI (Figure 1). An improvement after CGC was observed in 90.7% of these patients as assessed by TBGT and in 82.7% as indicated by BI respectively. A worsening in balance and gait ability was found in, respectively, 1.6% and in 9.2% of patients who underwent basic ADL (Figure 2). Better absolute scores in both tests (TGBG and BI) were noted in both patients with and without fracture after CGC; the results are summarized in Table 3.

In detail, a TBGT improvement of >5 was observed in 47.3% of the patients with complete TBGT assessment and was associated with female sex, a lower BI, and a worse score in TUG on admission (female sex: 82.2% versus 68.4%, *p* = 0.015; BI: median 40 (IQR: 30–50) versus 45 (IQR: 30–55), *p* = 0.010; TUG: median 5 (IQR: 4–5) versus median 4 (IQR: 3–5), *p* = 0.001)) (Table 4).

A BI improvement of ≥15 was documented in 63.0% of those patients who underwent a complete BI assessment and was associated with lower frequencies of diabetes mellitus and previous stroke (diabetes mellitus: 18.4% versus 32.4%, *p* = 0.009; previous stroke: 6.7% versus 14.3%, *p* = 0.035), but with higher frequencies of osteoporosis (22.9% versus 11.4%, *p* = 0.018) (Table 3). A BI improvement of ≥15 was also associated with a better TUG and a higher MMSE score on hospital admission (TUG: median 4 (IQR: 3–5) versus median 5 (IQR: 4–5), *p* = 0.001; MMSE: median 25 (IQR: 18–28) versus 18 (IQR: 0–27), *p* = 0.001) (Table 5).

## 4. Discussion

After completing CGC, 90.7% of patients with fractures in our study improved their balance and gait and 82.7% experienced an improvement in basic activities of daily living. Improvements in basic ADL were observed in individuals with better baseline mobility and gait and balance and higher MMSE scores, and improvements in gait and balance were associated with the factors of female sex and worse mobility and ability in gait and balance prior to CGC.

There are a variety of factors that seem to influence the outcome after CGC in older patients who have suffered a fracture. In the present investigation, an improvement in gait and balance was associated with the factor of female sex. By contrast, some authors reported no relevance for the factor of sex when investigating the effect of rehabilitation measures after hip fractures [29]; others indicate that males are more likely to benefit from rehabilitation than females [30]. However, in line with our results and with the majority of previous investigations, the factor of female sex seems to be of relevance with regard to improvements in balance and gait after CGC. For example, this finding is also in line with results obtained by Prestmo et al., who identified a more pronounced benefit in females after rehabilitation following hip fracture [31]. However, interpreting our results in the context of previous investigations, there is no plausible explanation for sex-related differences in outcome after fracture and subsequent CGC. Therefore, further research is warranted to assess differences between elderly women and men and in order to determine the benefit of CGC strategies for older patients and especially to identify sex-related parameters which have an impact on outcome.

In our particular group of patients, improvements in gait and balance were also associated with worse initial mobility and poor ADL, as indicated by the Tinetti assessment itself as well as the TUG test and the Barthel index. Our data, collected in a clinical setting in contrast to previous investigations, might unveil other factors that could be associated with a favorable outcome [30]. Our findings indicate that older patients with a recent fracture and an initially poor gait and balance performance (median Tinetti score of 8 (IQR 1–14)) might benefit most from CGC. The expected improvement is considerable; in our study, these patients improved their ability for performing gait and balance tasks by a median value of 14 (IQR 8–19) in the Tinetti score.

By contrast, better performances in balance and gait and walking ability prior to CGC facilitated greater improvements in basic activities of daily living. Comparable findings were observed regarding cognitive status; better MMSE scores prior to CGC were associated with favorable ADL outcomes after CGC. These findings indicate that improvements in activities of daily living in older patients with a recent fracture are dependent on a certain level of cognition, walking ability, and gait and balance. This finding is in line with previous investigations which indicated a direct relationship between the general capability for rehabilitation and the preexisting mobility and cognition status [32,33]. In conclusion, among older patients with a current fracture or fall event undergoing CGC, those who had performed well in terms of gait and balance (Tinetti score of median 10 (IQR 4–15)) and who had a good cognition status (MMSE score of median 25 (18–28)) could be expected to benefit most from the treatment.

An improvement in basic ADL by a minimum difference of 15 points in the Barthel index test prior versus after CGC was negatively associated with the presence of diabetes mellitus and previous stroke. Both parameters could be interpreted as markers for morbidity and are therefore related to higher grades of frailty in elderly patients, explaining their negative effect on recovery after CGC in older patients with fractures [34,35]. By contrast, in the present study, patients with osteoporosis seem to benefit more from CGC than those without. It could be speculated that coping with this disease in the context of a recent fall might increase the overall awareness of fall hazards, facilitating more significant improvements during recovery.

The strengths of the present investigation are the large number of participants who received CGC according to standardized protocols and the detailed documentation of all relevant parameters in the clinical process. However, there are also a number of limitations that must be taken into account. The major limitation of the study is that no control group with regular subject-specific treatment was available. Furthermore, it is possible that selection bias occurred because the patients selected for CGC during the geriatric pre-assessment were those who might profit most. Minor improvements in single TBGT and BI items are not addressed because of the decision to assess parameters based on a minimum pre-defined clinically relevant improvement of 5 points in TBGT and 15 points in BI.

Our results indicate that CGC is of great benefit to elderly patients after suffering a fracture, especially regarding the improvement of balance and gait and basic ADL and reveal potential determining factors for favorable outcomes. While greater improvements in daily activities were more likely in patients with a better previous gait and balance, walking ability and cognitive status, those with poorer walking ability and poorer gait and balance tended to achieve a better outcome with regard to gait and balance. Although our data are derived from clinical settings and are therefore more prone to differential bias, they elucidate the benefits of CGC under real-world conditions. From the clinician’s perspective, a selection bias is inevitable for the procedure.

## 5. Conclusions

When offered in specialized geriatric units, CGC improves the balance and gait and the basic ADL in elderly patients who have suffered a fracture. The factor of female sex was associated with improvements in balance and gait, while better baseline mobility and cognitive status facilitate a better outcome with regard to activities of daily living.

## Figures and Tables

**Table 1:** Treatment methods included in comprehensive geriatric care.

Treatment Method	Measures/Target Deficits/Symptoms
Physiotherapy	general mobilization gait training physical therapy musculoskeletal therapy prophylaxis treatment of contractures
Occupational therapy	exercises focusing on activities of daily living (such as food intake or independent dressing)
Speech therapy/orofacial therapy	treatment of dysarthria/aphasia treatment of dysphagia *
Psychological support	supportive measures (e.g., talking therapy) motivation

* includes the assessment of and therapy for swallowing.

**Table 2:** Patients treated in the geriatric unit.

	Total Group (*n* = 1099)	Fractures (*n =* 300)	No Fractures (*n* = 799)	p *
Age (median, IQR *, years)	83.1 (79.0–87.8)	85.6 (81.1–89.6)	82.4 (78.3–86.9)	0.001
Age ≥ 80 years	754 (68.6%)	239 (79.7%)	515 (64.5%)	0.001
Sex				
female	704 (64.1%)	219 (73.0%)	314 (60.7%)	0.001
male	395 (35.9%)	81 (27.0%)	314 (39.3%)	
Co-morbidities				
Hypertension	853 (77.6%)	246 (82.0%)	607 (76.0%)	0.035
Diabetes mellitus	337 (30.7%)	69 (23.0%)	268 (33.5%)	0.001
Heart failure	258 (23.5%)	66 (22.0%)	192 (24.0%)	0.523
Coronary heart disease	281 (25.6%)	61 (20.3%)	220 (27.5%)	0.016
Peripheral artery disease	59 (5.4%)	9 (3.0%)	50 (6.3%)	0.035
Atrial fibrillation	388 (35.3%)	93 (31.0%)	295 (36.9%)	0.076
Functional assessment on admission				
Barthel index (median, IQR)	45 (30–60)	40 (30–50)	45 (30–60)	0.001
Tinetti geriatric assessment (median, IQR)	11 (12–16)	8 (1–14)	12 (4–17)	0.001
Geriatric depression scale (median, IQR)	3 (1–6)	3 (1–6)	3 (1–6)	0.844
Geriatric depression scale > 5	302 (27.7%)	83 (27.8%)	219 (27.6%)	0.999
Timed up and go (median, IQR)	4 (3–5)	5 (3–5)	4 (3–5)	0.001
MMSE (median, IQR) (n = 812)	26 (21–28)	25 (19–28)	26 (21–28)	0.282

* refers to interquartile range.

**Table 3:** Tinetti score and Barthel index; values for geriatric patients with and without fractures prior to versus after comprehensive geriatric care.

	Prior to CGC *	After CGC *	p *
Patients with fracture			
Tinetti score (median, IQR)	8 (1–14)	14 (8–19)	<0.001
Barthel index (median, IQR)	40 (30–50)	55 (40–75)	<0.001
Patients without fracture			
Tinetti score (median, IQR)	12 (4–17)	16 (9–21)	<0.001
Barthel index (median, IQR)	45 (30–60)	60 (45–80)	<0.001

* refers to comprehensive geriatric care.

**Table 4:** Factors associated with an improvement of >5 in Tinetti score in elderly patients with fractures after comprehensive geriatric care.

	Total Group (n = 258)	Improvement in Tinetti Score > 5 (n = 122)	No Improvement in Tinetti Score > 5 (n = 136)	p *
Age (median, IQR, years)	85.5 (81.1–89.9)	85.7 (81.6–89.9)	84.7 (80.9–88.9)	0.368
Age ≥ 80 years	208 (80.6%)	102 (83.6%)	106 (77.9%)	0.273
Sex				
female	193 (74.8%)	100 (82.2%)	93 (68.4%)	0.015
male	65 (25.2%)	22 (18.0%)	43 (31.6%)	
Co-morbidities				
Hypertension	209 (81.0%)	103 (84.4%)	206 (77.9%)	0.206
Diabetes mellitus	56 (21.7%)	35 (28.7%)	21 (15.4%)	0.011
Heart failure	53 (20.5%)	20 (16.4%)	33 (24.3%)	0.126
Renal insufficiency	73 (28.3%)	34 (27.9%)	39 (28.7%)	0.891
Coronary heart disease	49 (19.0%)	22 (18.0%)	27 (19.9%)	0.752
Peripheral artery disease	8 (3.1%)	3 (2.5%)	5 (3.7%)	0.726
Atrial fibrillation	78 (30.2%)	35 (28.7%)	43 (31.6%)	0.684
Chronic pulmonary artery disease	13 (5.0%)	5 (4.1%)	8 (5.9%)	0.579
Dementia	61 (23.6%)	32 (26.2%)	29 (21.3%)	0.381
Morbus Parkinson	14 (5.4%)	4 (3.3%)	10 (7.4%)	0.177
Previous stroke	24 (9.3%)	13 (10.7%)	11 (8.1%)	0.524
Osteoporosis	51 (19.8%)	29 (23.8%)	22 (16.2%)	0.159
Vitamin B deficiency	133 (51.6%)	60 (49.2%)	73 (53.7%)	0.533
Location of fractures				
Lower extremities	143 (55.4%)	73 (59.8%)	70 (51.5%)	0.236
Pelvic region	38 (14.7%)	21 (17.2%)	17 (12.5%)	
Spinal column	36 (14.0%)	13 (10.7%)	23 (16.9%)	
Thorax	7 (2.7%)	2 (1.6%)	5 (3.7%)	
Upper extremities	24 (9.3%)	8 (6.6%)	16 (11.8%)	
Different locations	10 (3.9%)	5 (4.1%)	5 (3.7%)	
Short term adverse events while hospitalization				
Diffuse pain	84 (32.6%)	45 (36.9%)	39 (28.7%)	0.184
Delirium	9 (3.5%)	5 (4.1%)	4 (2.9%)	0.739
Pneumonia	15 (5.8%)	7 (5.7%)	8 (5.9%)	0.999
Urinary tract infection	40 (15.5%)	24 (19.7%)	16 (11.8%)	0.087
Dizziness	13 (5.0%)	7 (5.7%)	6 (4.4%)	0.777
Deep vein thrombosis	1 (0.4%)	0 (0%)	1 (0.7%)	0.999
Pulmonary emboli	1 (0.4%)	0 (0%)	1 (0.7%)	0.999
Electrolyte imbalance	75 (29.1%)	33 (27.0%)	42 (30.9%)	0.583
Hypokalemia	61 (23.6%)	26 (21.3%)	35 (25.7%)	0.464
Hyponatremia	20 (7.8%)	10 (8.2%)	10 (7.4%)	0.820
Functional assessment on admission				
Barthel index (median, IQR)	40 (30–50)	40 (30–50)	45 (30–55)	0.010
Tinetti on admission (median, IQR)	8 (1–14)	5 (0–11.25)	12 (7.25–18)	0.001
Geriatric depression scale (median, IQR)	3 (1–6)	4 (1–6)	3 (1–5)	0.195
Geriatric depression scale >5	67 (26.0%)	37 (30.3%)	30 (22.1%)	0.155
Timed up and go (median, IQR)	5 (3–5)	5 (4–5)	4 (3–5)	0.001
MMSE (median, IQR)	24 (10–27)	24 (17.75–27)	25 (13.3–28)	0.599
Discharging mode				
Home care	255 (98.8%)	120 (98.4%)	135 (99.3%)	0.604
Referral to other department	3 (1.2%)	1 (1.6%)	2 (0.7%)	

* refers to interquartile range.

**Table 5:** Factors associated with an improvement of ≥15 in the Barthel index in elderly patients with fractures after comprehensive geriatric care.

	Total Group (n = 284)	Improvement in Barthel Index ≥ 15 (n = 179)	No Improvement in Barthel Index ≥ 15 (n = 105)	p *
Age (median, IQR, years)	85.6 (81.1–89.9)	84.5 (81.6–89.8)	86.4 (81.2–90.7)	0.332
Age ≥ 80 years	226 (79.6%)	139 (77.7%)	87 (82.9%)	0.361
Sex				
female	210 (73.9%)	137 (76.5%)	73 (69.5%)	0.209
male	74 (26.1%)	42 (23.5%)	32 (30.5%)	
Co-morbidities				
Hypertension	231 (81.3%)	149 (83.2%)	82 (78.1%)	0.344
Diabetes mellitus	67 (23.6%)	33 (18.4%)	34 (32.4%)	0.009
Heart failure	61 (21.5%)	38 (21.2%)	23 (21.9%)	0.882
Renal insufficiency	82 (28.9%)	46 (25.7%)	36 (34.3%)	0.137
Coronary heart disease	56 (19.7%)	39 (21.8%)	17 (16.2%)	0.282
Peripheral artery disease	9 (3.2%)	6 (3.4%)	3 (2.9%)	0.999
Atrial fibrillation	88 (31.0%)	52 (29.1%)	36 (34.3%)	0.356
Chronic pulmonary artery disease	16 (5.6%)	7 (3.9%)	9 (8.6%)	0.114
Dementia	75 (26.4%)	45 (25.1%)	30 (38.6%)	0.578
Morbus Parkinson	18 (6.3%)	9 (5.0%)	9 (8.6%)	0.313
Previous stroke	27 (9.5%)	12 (6.7%)	15 (14.3%)	0.035
Osteoporosis	53 (18.7%)	41 (22.9%)	12 (11.4%)	0.018
Vitamin B deficiency	145 (51.1%)	91 (50.8%)	54 (51.4%)	0.999
Location of fractures				
Lower extremities	159 (50.0%)	97 (54.2%)	62 (59.0%)	0.606
Pelvic region	44 (15.5%)	32 (17.9%)	12 (11.4%)	
Spinal column	40 (14.1%)	24 (13.4%)	16 (15.2%)	
Thorax	6 (2.1%)	3 (1.7%)	3 (2.9%)	
Upper extremities	24 (8.5%)	17 (9.5%)	7 (6.7%)	
Different locations	11 (3.9%)	6 (3.4%)	5 (4.8%)	
Short term adverse events while hospitalization				
Diffuse pain	91 (32.0%)	63 (35.2%)	28 (26.6%)	0.149
Delirium	12 (4.2%)	7 (3.9%)	5 (4.8%)	0.765
Pneumonia	21 (7.4%)	11 (6.1%)	10 (9.5%)	0.349
Urinary tract infection	46 (16.2%)	30 (16.8%)	16 (15.2%)	0.868
Dizziness	15 (5.3%)	11 (6.1%)	4 (3.8%)	0.584
Deep vein thrombosis	1 (0.4%)	0 (0%)	1 (1.0%)	0.370
Pulmonary emboli	1 (0.4%)	1 (0.6%)	0 (0%)	0.999
Electrolyte imbalance	89 (31.3%)	53 (29.6%)	36 (34.3%)	0.429
Hypokalemia	75 (26.4%)	44 (24.6%)	31 (29.5%)	0.404
Hyponatremia	23 (8.1%)	14 (7.8%)	9 (8.6%)	0.825
Functional assessment on admission				
Barthel index (median, IQR)	40 (30–50)	40 (30–55)	35 (20–50)	0.289
Tinetti on admission (median, IQR)	8 (1–14)	10 (4–15)	4 (0–11)	0.001
Geriatric depression scale (median, IQR)	3 (1–6)	4 (1–6)	3 (0–6)	0.180
Geriatric depression scale >5	77 (27.1%)	52 (29.1%)	25 (23.8%)	0.407
Timed up and go (median, IQR)	5 (3–5)	4 (3–5)	5 (4–5)	0.001
MMSE (median, IQR)	24 (10–27)	25 (18–28)	18 (0–27)	0.001
Discharging mode				
Home care	282 (99.3%)	177 (98.9%)	105 (100%)	0.532
Referral to other department	2 (0.7%)	2 (1.1%)	0 (0%)	

* refers to interquartile range.

## Data Availability

Data is available upon reasonable request by contacting the corresponding author.
